# Supplementary material for: Radar near-field sensing using metasurface for biomedical applications
Source: Commun Eng. 2024 Mar 19;3:51. doi: 10.1038/s44172-024-00194-4 (PMC10955967; doi:10.1038/s44172-024-00194-4)
Supplement: Supplementary file 2 — Supplementary Information [file 44172_2024_194_MOESM2_ESM.pdf]

Supplementary Information for: Radar Near-Field Sensing Using Metasurface for Biomedical Applications

Mohammad Omid Bagheri[1,*], Ali Gharamohammadi[1], Serene Abu-Sardanah[1], Omar M Ramahi[1], and George Shaker[1]

[1]Department of Electrical and Computer Engineering, University of Waterloo, Waterloo, ON, Canada
[*]Corresponding Author: Mohammad Omid Bagheri, Email: omid.bagheri@uwaterloo.ca

Fabrication process and measurement setup

In this section, the fabrication process of the designed transmitarray metasurface and the required measurement setup for measuring the near-field power inside the proposed phantom, beaker filled with pure water, and power reflected to the radar system using radar signal processing are presented and discussed.

Supplementary Note 1: Fabrication setup and considerations

Regarding the metasurface fabrication process, the three steps are required as the following: i) the transmitarray metasurface using printed circuit technology consisting of the phase-compensated two-layer unitcells to realize a low-profile square lattice array with total dimensions of $1.54\lambda\times1.54\lambda$ printed on both sides of a low-loss dielectric substrate, Rogers-RO4003, with the thickness of 0.8 mm. As shown in Fig. 8c, the metasurface is fabricated at the same dimensions as the radar for full integration. The designed array part of the metasurface also covers the radar antenna to provide focused power into the water medium. ii) a dielectric fixture using 3D printing technology to stabilize the metasurface at half-wavelength air gap distance above the radar system as shown in Fig. 8c; iii) a dielectric fixture required to be designed and fabricated using 3D printing technology for the measurement setup to make the prob coming out from the Spectrum Analyzer stable inside the beaker-filled with water proving high repeatability in the measurement results of the transferred power as shown in Fig. 8d. Furthermore, the dimensions of the measurement dielectric fixture are adjusted to tightly enclose the radar dielectric housing and maintain the position of the beaker at the center of the array.

In the main manuscript, two sets of measurement processes are discussed. Ensuring precision in the results necessitates careful consideration of the signal processing configuration for the 60 GHz FMCW Infineon radar system, as described in the following note.

Supplementary Note 2: Radar signal processing

FMCW radar systems broadcast an amplified and frequency-modulated electromagnetic signal produced by a signal generator into the environment and then receive the reflected signals from various objects which carry properties such as range and radar cross-section.

Supplementary Fig. S1a shows a common block diagram of the current off-the-shelf FMCW radar modules where both transmitter and receiver are at the same place. As shown, after amplification of the received signal by a low noise amplifier (LNA), the mixer correlates the transmitted and received signals leading to the production of low-frequency and high-frequency signals. The low-pass filter is added to the diagram to filter low-frequency signals in the next block, and then an analog-to-digital converter (ADC) is used to convert it to a digital signal which can be processed based on the given information of the designed signal, especially frequency bandwidth.

The signal generator in FMCW radar sweeps in a range of frequency (f_{min} to f_{max}) linearly with a positive slope of K and a time duration of T and prepares the output signal which is called chirp. The resulted frequency bandwidth, BW, for a chirp is as follows,

$$\text{BW} = f_{\text{max}} - f_{\text{min}} = K \times T \tag{S1}$$

The frequency bandwidth presented in (S1) determines the range resolution of the radar. The relationship between range resolution and frequency bandwidth as presented in (S2) shows that the radar with higher frequency bandwidth provides better range resolution.

$$\Delta R = \frac{C}{2\,\text{BW}} \tag{S2}$$

Where ΔR is range resolution and C is light velocity in the free space. The range resolution in (S2) discretizes the range of the FMCW radar such that better range resolution provides accurate range estimation and better discrimination between two close reflections. The range in FMCW radar can be estimated from the peak frequency of the reflected chirp signal in the frequency domain by taking Fast Fourier Transform (FFT) of the chirp signal in the time domain that is assumed as (S3). Suppose that a single reflection with a delay of t_{d} from the environment, results in (S4) at the output of the mixer shown in Supplementary Fig. S1a.

$$x(t) = A\ \cos\left(2\pi f_{\text{min}} t + \pi K t^2\right), \quad 0 < t < T \tag{S3}$$

$$x(t) = A\ \cos\left(2\pi f_{\text{min}} t + \pi K t^2\right) \times A\ \alpha \cos\left(2\pi f_{\text{min}}(t - t_{\text{d}}) + \pi K (t - t_{\text{d}})^2\right) \tag{S4}$$

Where α corresponds to the effect of environment and target on the transmitted signal amplitude. By passing through the low pass filter shown in Supplementary Fig. S1a, the resulting signal which is called the beat signal is as (S5). Considering the low range of the target, t_{d} is much less than t in (S5) and the beat signal can be simplified as (S6). Replacing $t_{\text{d}} = 2R/C$, the relationship between the beat and the range of the target, R, can be obtained as (S7). By taking the Fast Fourier Transform from the signal presented in (S7), the range of the target can be determined.

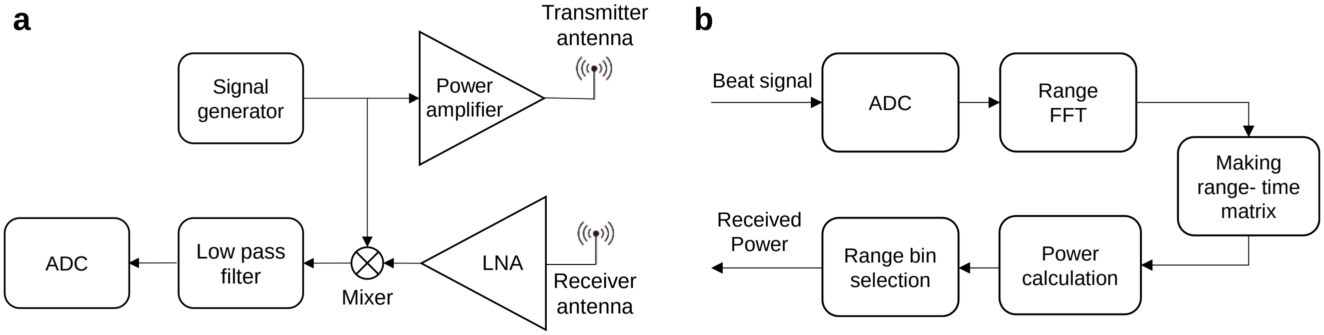

Supplementary Fig. S1 (a) A simple block diagram of radar before signal processing where both transmitter and receiver are at the same place. (b) The signal processing chain for power calculation

$$x(t) = A^2\,\alpha\cos\left(2\pi f_{\min}t_{\mathrm{d}} + 2\pi K(t\,t_{\mathrm{d}} - {t_{\mathrm{d}}}^2)\right) \tag{S5}$$

$$x(t) \simeq A^2\,\alpha\cos\left(2\pi f_{\min}t_{\mathrm{d}} + 2\pi K\,t\,t_{\mathrm{d}}\right) \tag{S6}$$

$$x(t) \simeq A^2\,\alpha\cos\left(\frac{4\pi R}{C}(f_{\min} + Kt)\right) \tag{S7}$$

In FMCW radar, the range resolution along with chirp length determines the maximum range of the system. The chirp length can be optimized to remove environmental clutter. Although the near-field measurement requires monitoring the low ranges, more samples benefit from clutter reflection removal. The extra range samples receive the close clutter effects up to the maximum range of the system and then these samples can be removed. In addition, the finer range resolution provides more accurate details of the environment. In this study, the best achievable range resolution of the employed system, 3 cm, is applied for signal design. By considering this range resolution and the maximum range of the system equal to 1 m, the chirp length, M, can be determined using (S8) as equal to 64.

$$M = \frac{R_{\max}}{2\,\Delta R} \tag{S8}$$

Using (S1) to (S8), the designed signal parameters are summarized in Supplementary Table S1 which are used in the signal processing chain. By considering the Pulse Repetition Frequency (PRF) equal to 20 and the sampling frequency of the beat signal equal to 1 MHz, the chirp slope, CS, is obtained as 78.128 MHz/µs which can be supported by the employed system.

Supplementary Fig. S1b depicts the signal processing chain for power calculation in a specific range bin. After ADC, the spectrum of the beat signal, which has peaks determining the targets at various ranges, is obtained by applying an FFT. This FFT can also be considered a range FFT since it reveals a range of reflections. Each range FFT bin corresponds to a range interval discretized by range resolution. For instance, if the range resolution is 3 cm and a target range

Table S1: The designed signal parameters

Parameter	Quantity	Value
PRF	pulse repetition frequency	20
BW	signal chirp bandwidth	5 GHz
ΔR	radar range resolution	3 cm
$R_{\max}$	maximum range of system	96 cm
M	chirp length	64
f_s	beat signal sampling frequency	1 MHz
CS	chirp slope	78.128 MHz/µs

Algorithm S1 Power Calculation Algorithm

Input: Range-time Matrix in the Frequency Domain and Selected Range Bin
Output: Power Calculation
counter = 0
Sum = 0
for i in range (1, N): **do**
    counter = counter + 1
    selected sample = Beat signals in the frequency domain (selected range bin)
    sum = sum + selected sample
    Power = sum / counter
    Return Power

is 20 cm, this target will appear in the 7th range bin. By collecting N consecutive chirps over time and putting them into a matrix, the range-time matrix is created. This matrix has M rows corresponding to all numbers of range bins and N columns corresponding to the number of chirps. Then, the desired range based on the experiment is selected and the power is calculated for that range bin. Supplementary Algorithm S1 calculates power for a range-time matrix in a determined range.
